# Barriers and Facilitators to Teledermatology and Tele-Eye Care in Department of Veterans Affairs Provider Settings: Qualitative Content Analysis

**DOI:** 10.2196/50352

**Published:** 2024-02-07

**Authors:** Yiwen Li, Charlene Pope, Jennifer Damonte, Tanika Spates, April Maa, Suephy Chen, Howa Yeung

**Affiliations:** 1 Department of Dermatology Emory University School of Medicine Atlanta, GA United States; 2 Charleston Veterans Affairs Health Equity and Rural Outreach Innovation Center Ralph H. Johnson Veterans Affairs Health Care System Charleston, SC United States; 3 Clinical Resource Hub Veterans Affairs Veterans Integrated Service Network 7 Atlanta, GA United States; 4 Department of Ophthalmology Emory University School of Medicine Atlanta, GA United States; 5 Department of Dermatology Duke University School of Medicine Durham, NC United States

**Keywords:** telemedicine, dermatology, eye, implementation science, stakeholder participation, veterans’ health

## Abstract

**Background:**

Veterans Affairs health care systems have been early adopters of asynchronous telemedicine to provide access to timely and high-quality specialty care services in primary care settings for veterans living in rural areas. Scant research has examined how to expand primary care team members’ engagement in telespecialty care.

**Objective:**

This qualitative study aimed to explore implementation process barriers and facilitators to using asynchronous telespecialty care (teledermatology and tele-eye care services).

**Methods:**

In total, 30 participants including primary care providers, nurses, telehealth clinical technicians, medical and program support assistants, and administrators from 2 community-based outpatient clinics were interviewed. Semistructured interviews were conducted using an interview guide, digitally recorded, and transcribed. Interview transcripts were analyzed using a qualitative content analysis summative approach. Two coders reviewed transcripts independently. Discrepancies were resolved by consensus discussion.

**Results:**

In total, 3 themes were identified from participants’ experiences: positive perception of telespecialty care, concerns and challenges of implementation, and suggestions for service refinement. Participants voiced that the telemedicine visits saved commute and waiting times and provided veterans in rural areas more access to timely medical care. The mentioned concerns were technical challenges and equipment failure, staffing shortages to cover both in-person and telehealth visit needs, overbooked schedules leading to delayed referrals, the need for a more standardized operation protocol, and more hands-on training with formative feedback among supporting staff. Participants also faced challenges with appointment cancellations and struggled to find ways to efficiently manage both telehealth and in-person visits to streamline patient flow. Nonetheless, most participants feel motivated and confident in implementing telespecialty care going forward.

**Conclusions:**

This study provided important insights into the positive perceptions and ongoing challenges in telespecialty care implementation. Feedback from primary care teams is needed to improve telespecialty care service delivery for rural veterans.

## Introduction

**Figure 1:**
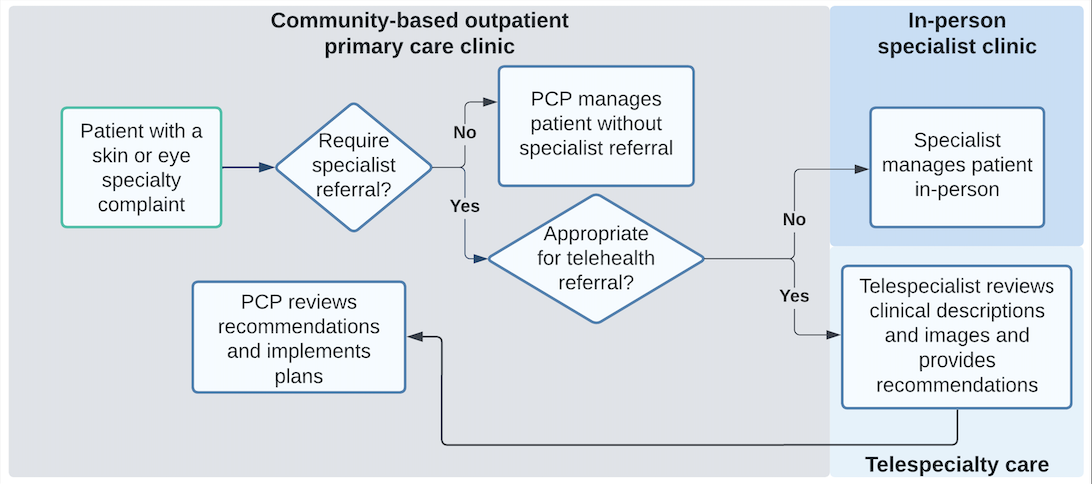
Asynchronous telehealth referral flowchart. In-patient primary care evaluation determines patient management by the PCP or referral to a specialist. If a specialist is needed, the PCP decides between in-person specialist clinic or telespecialty care. For telespecialty care, clinical data and images are sent to the telespecialist for analysis. The results are then communicated by the PCP to the patient, speeding up further treatment processes. PCP: primary care physician.

The use of telemedicine has been steadily increasing and has expanded rapidly during the COVID-19 pandemic [1]. Veterans’ health care systems have been early adopters of asynchronous telemedicine, also known as the store-and-forward mode of consultation and sometimes referred to as “eConsult” or “eTriage.” In this approach, a brief clinical history and images are collected during an in-person primary care visit at a community-based outpatient clinic. These records are subsequently transmitted to telespecialists at a distant site for evaluation, and the results are communicated to the patient by the referring primary care provider (Figure 1). Patients with additional needs are identified through this process for expedited treatment. In this manner, veterans are provided timely access to high-quality specialty care services in primary care settings, especially in rural areas [2].

With an emphasis on visual diagnosis, asynchronous telemedicine is well-suited for Teledermatology and Technology-Based Eye Care Services [3]. However, concerns have been raised to adopt telemedicine for specialty care on a larger scale, as certain sites may be disadvantaged with the lack of clinical resources and administrative experience in implementing complex programs. The goal of this qualitative study is to better understand implementation process facilitators and barriers to telemedicine use for specialty care.

## Methods

### Study Design

Between October 2, 2020, and January 31, 2021, we conducted in-depth interviews with primary care providers, nurses, telehealth clinical technicians, medical and program support assistants, and administrators from 2 community-based outpatient clinics and distant reading sites. Semistructured interviews (interview guide detailed in Textbox 1) aimed to explore perspectives, identify telespecialty care facilitators and barriers, and derive solutions from community-based outpatient clinics [4]. A trained interviewer (TS) conducted 30 individual telephone interviews lasting 30-60 minutes, with digital audio recording and participant consent. Interviews were transcribed professionally and deidentified. Qualitative content analysis followed a summative approach [5] with latent content analysis for underlying meanings and patterns. Coders (CP and JD) independently reviewed transcripts, resolving discrepancies by consensus. Diagramming mapped conceptual relationships across stakeholder perceptions to identify facilitators, barriers, and solutions.

**Qualitative interview questions.**

Q1. What percentage of your time is dedicated to TECS (Technology-Based Eye Care Services) or TD (Teledermatology), as compared to face-to-face care?

Q2. How do you feel about TECS or TD at our location?

Q3. How motivated or committed do you feel your site is in implementing TECS or TD?

Q4. How ready do you feel your site is, to implement TECS or TD?

Q5. What has been your experience in working with the regional telehealth service reading hub, in which veterans’ images taken at your CBOC (community-based outpatient clinic) or site are interpreted by a clinician outside of your site?

Q6. What worked well in facilitating implementation of TECS or TD at your site?

Q7. What types of data or reports were helpful in facilitating implementation of TECS or TD at your site?

Q8. What issues or barriers have you experienced in implementing TECS or TD at your site?

Q9. What have been some unintended consequences following implementation of TECS or TD at your site?

Q10. How were challenges in implementation of TECS or TD managed at your site?

Q11. What changes do you recommend in sustaining TECS or TD at your site?

Q12. What recommendations would you offer to other CBOC sites providing, or considering providing, TECS or TD?

Q13. Please share any additional thoughts or information that you would like us to know.

### Ethical Considerations

This study received approval from the Emory University Institutional Review Board (STUDY00000383) on June 3, 2020, and from the Atlanta Veterans Affairs (VA) Medical Center Research and Development Committee. The results are reported in accordance with COREQ (Consolidated Criteria for Reporting Qualitative Research) guidelines [6]. All participants provided verbal informed consent prior to the study conduct, and participant data were deidentified. No compensation was provided to the participants.

## Results

### Overview

Of the 30 participants, a total of 27 (90%) had experienced the hybrid format of telespecialty encounters, where patients visited the community-based outpatient clinic site for image acquisition during the study period, and 3 (10%) had in-person visits only. Interviews identified two primary facilitators to telespecialty care: (1) positive perception of telespecialty care and (2) optimized implementation processes (task lists, deadlines, and bringing together multiple capable diverse stakeholders in regular meetings).

### Positive Perception of Telespecialty Care

#### Overview

Stakeholders from various roles provided insights indicating positive telespecialty care experiences reported by veterans (domain I). Telemedicine saved commute and waiting times, enhancing access for rural veterans (domain II). The telespecialty care programs are regarded to provide good quality of care (domain III). Telespecialty care increased resources for routine care, saved appointments at the main facility, and allowed VA health care to receive more workload credit (domain IV).

#### Domain I: Patient Satisfaction

Patient satisfaction is the first domain emerging from interviews. Interviewees speculated about the reasons, but all stated that patients were satisfied with the services they received. One participant explained:

I don’t know if it’s because we’re more accessible right now, that may be the reason. But they all seem to be very satisfied with their care and feel like they’ve got a very good exam.

Another participant explained that:

[The patients] love this. Having the specialty Technology-based Eye Care Services and then being able to come to a clinic in their community to pick up their glasses, all their services they need for their eyes are done in one stop shop.

The interviews highlighted the patient satisfaction benefits of the Teledermatology and Technology-Based Eye Care Services programs.

#### Domain II: Access

##### Overview

Access was a frequently discussed domain in the interviews. Participants believed telespecialty care services increased veterans’ health care access, especially benefiting those in rural areas. Factors contributing to this enhanced access included travel (distance and time), timely care, and integration with primary care.

##### Travel

Telespecialty care programs at local community–based outpatient clinics offer improved health care access compared to traveling to a main VA facility with on-site specialty care. With more community-based outpatient clinic locations than main VA facilities, traveling distance and time for veterans are reduced. One participant highlighted the challenges and difficulties veterans faced when seeking specialty care.

Technology-based Eye Care Services is very helpful when it’s out in rural areas ... Dermatology as well. You know, I think a lot of times people go under the assumption that everybody is close by, that there’s a VA everywhere and that if there’s a VA in your community and your community is small enough for you to get to that VA within 30-45 minutes or maybe an hour. I think that having forward thinking or being very realistic would help because some people travel, you know, three or four hours, to get to their clinic.

Telespecialty care services provided at community-based outpatient clinic locations enable veterans to receive crucial care without burdensome travel.

##### Timely Care

The main VA medical centers provide various services from annual checkups to major surgeries. However, these centers frequently have long waitlists due to the limited providers and availabilities. Telespecialty care services at community-based outpatient clinics help reduce wait times and enable timely care. One interviewee stated that:

... the speed that we’re able to provide the care is better. So instead of waiting for the patient to have an appointment in a face-to-face grid with limited access, we’re using these technologies at all of our sites and the time that passes between the patient needing the care, the provider consulting for the care, and then receiving it, it decreases a great deal.

Further, wait times for community-based outpatient clinic appointments tend to be shorter than the main VA medical centers. One interviewee revealed:

... mostly about the technicians that they see, the fact that they were able to quickly get in and out. It wasn’t a long wait time for them. Usually with eye exams, they have to wait maybe between two and four hours when they go to the main hospital, so that’s a big plus.

##### Convenient Access Integration Into Primary Care or In-House Service

Another benefit that telespecialty care provides at community-based outpatient clinic locations is the ease of referrals from primary care providers. Community-based outpatient clinics provide the most common outpatient services (eg, primary care) and typically lack in-house specialty care providers. When patients require ophthalmology or dermatology referrals, they typically need to make an appointment at the main VA medical center community clinics. With telespecialty care programs at a community-based outpatient clinic, patients can often undergo specialty care imaging acquisition during the same visit as their primary care appointment.

One interviewee stated:

... from the Derm aspect. If it was something that, say, the primary physician sees while they’re there physically in the clinic or face-to-face, they can immediately put in a Teledermatology consult while the patient is at the clinic and the patient doesn’t have to come back for a second trip to the clinic.

This remote access reduces the burden on the patients for having to return to the clinic for follow-up care. One of the primary care physicians provided:

Many of our veterans did not want to travel the sometimes 40 to sometimes 1 ½ hour commute between traffic and the time of day. And so to be able to have a dermatology and ophthalmology consultation at the local site, was very convenient for the veteran population that we served.

The integration with primary care clinics at community-based outpatient clinics adds even more convenience to patients, and they can get “one-stop shop” health care.

#### Domain III: Quality of Care

Teledermatology and Technology-Based Eye Care Services provide telespecialty services with improved access while maintaining quality of care comparable to in-person care, meeting their goal of providing veterans with high-quality specialty care in a timely manner.

One interviewee pointed out:

... as far as [s/he] know[s] about it, it provides the same quality of care as a face-to-face visit would.

Other interviewers echoed this and stated:

I feel the quality of care is excellent.

One of the participants posits:

It would be nearly impossible for me to replicate the quality of care that I get from the Technology-based Eye Care Services.

#### Domain IV: Workload Credit

The last domain relates to how the telespecialty care programs benefit the VA. Teledermatology and Technology-Based Eye Care Services effectively triage patients into those who can be managed remotely, thereby freeing up appointments for patients needing face-to-face care. One interviewee explained that:

They were able to stream-line the process so that only those who have cancerous appearing lesions could be brought to the medical center and so therefore you were able to get to the greatest number of veterans that truly needed that service.

### Optimized Implementation Process

#### Overview

Communication process emerged as a central theme for successful telespecialty care program implementation. The implementation team’s engagement approach, communication, and availability at regular and frequent huddles to work through issues were viewed as important. The implementation team lead functioned as an ally and integrated as part of the site team. Clinical staff found communication between the site and the implementation team to be important. One interviewee stated:

Two-way communication on a day to day, week to week, month to month basis was very helpful.

Regularly scheduled meetings are a crucial aspect of implementing a successful program launch. By providing structure and opportunities to discuss progress and overcome barriers, these meetings are essential for tracking progress and achieving objectives. Key stakeholders from local VA departments participate in these meetings, ensuring that all necessary perspectives are considered. According to one interviewee, the involvement of stakeholders from different departments facilitates efficient communication and problem-solving:

It is good because it’s several different people from different locations that are tackling it. I feel like everybody that connects are different people, so if one person doesn’t know exactly who to speak to, someone else may know, and so we can get it done pretty quickly.

Regular engagement with stakeholders from various departments helps to streamline operations and minimize delays while also promoting collaboration and a shared sense of purpose. This can lead to increased efficiency as well as a greater focus on shared goals and objectives.

#### Concerns and Challenges of Implementation

Participants voiced concerns about technical challenges, staffing shortages to cover both in-person and telehealth visit needs, overbooked schedules leading to delayed referrals, the need for a more standardized operation protocol, and more hands-on training with formative feedback among supporting staff. Participants also faced challenges with appointment no-shows and last-minute cancellations and struggled to find ways to efficiently manage both telehealth and in-person visits to streamline patient flow.

#### Domain I: Staffing

Staffing presented a barrier when sites were limited in size and limited trained telehealth clinical technicians or their turnover, as these 2 participants portrayed:

My facility is a small community-based outpatient clinic and we have four nurses and four nurses are required to triage patients and we’re going to have to have one of those nurses dedicated to doing the tele-imaging, then that’s going to be a barrier for that clinic.

I feel like we need to have more imagers trained. So basically, if someone calls in sick, Teledermatology just shuts down. We have to have a backup plan.

#### Domain II: Scheduling

Participants described difficulty with scheduling due to telespecialty care appointment cancellations:

Because a clinic has been canceled so many times due to equipment failure and patients being rescheduled, it kind of clogged up the availability, you know, it ran availability out more than 30 days, so a patient is not able to get to a clinic that’s close by them at times so there was an issue, or there is an issue with that. That’s an ongoing issue with Technology-based Eye Care Services.

Among the unintended consequences of the implementation of Teledermatology and Technology-Based Eye Care Services was the additional time required for scheduling, as extra visits were added for referrals from primary care.

It did impact face-to-face care from a primary care perspective because we were the face for Teledermatology and Technology-based Eye Care Services ... the prerequisite is that the primary care physicians were the ones who were submitting the consults. So, it required us to at least see that’s going on. And so, it was an additional visit with us that we had to fit in outside of maybe a normally scheduled primary care visit.

At the heart of scheduling, a technician advised that referrals of complex patients with multiple morbidities can be a barrier:

Not everybody is a candidate for the program; If they have multiple diseases, if they have certain levels of complications, they’re not suited for the Technology-based Eye Care Services program, and they shouldn’t be scheduled because then they wait to see you and then they’ve got to wait to see somebody else because you couldn’t do what they needed to have done. So, there are several little things that can really wreak havoc on a day and on a schedule.

#### Domain III: Equipment

Equipment failure was seen as increasing wait times, causing appointment cancellations and rescheduling. One huge barrier was streamlining the reporting process for equipment failure, involving cameras, computers, dermatoscopy, and nonmydriatic fundus photography equipment. With equipment failure or technician absence, veteran care was canceled, and no accurate estimation could be given to schedule the next available appointment, as illustrated by this participant:

And equipment failure leads to wait time, longer wait times, and patients having to be cancelled and rescheduled, and a lot of times these patients are coming from, you know, 30, 40, 50, 70 miles away. So when you have to push back their appointment time or cancel it altogether, it gets very frustrating for the veterans and for the technicians.

#### Domain IV: Protocol

Participants also observed a need for specific personnel delegation and a standard operating protocol in place for troubleshooting.

There is a need. It’s a great program, but that way, no matter what role you’re in, and if you get looped in, you know, if you don’t have the key people in place, you might just have something to go by, just like a checklist, would be my only recommendation as far as that goes.

#### Domain V: Training

Training was identified as an ongoing need that affects service provision. Training needs to concentrate on orienting staff at all levels, including those not directly performing Teledermatology and Technology-Based Eye Care Services on the scope of telespecialty care practices. The awareness of the programs will enable them to make the best use of the services, as this technician describes:

... because the Technology-based Eye Care Services program is a new way of providing eye care, and the other departments not really being familiar with what we do, there was a period of months where it took, I felt like longer, than expected to help the staff understand what we provided.

## Discussion

### Principal Findings

This study identified facilitators and potential challenges to telespecialty care implementation through summative content analysis, highlighting the complexity of telespecialty care as an intervention to bridge the access issue for veterans. In line with these findings, recommendations provided in Textbox 2 further complement this study, offering actionable steps for improving the implementation of telespecialty care.

**Telespecialty care implementation recommendations.**

**Staffing**

- Ensure telespecialty care technicians are not simultaneously assigned regular clinical duties.
- Train additional telehealth technicians and standardize backup plans for staff absences.

**Scheduling**

- Implement real-time scheduling to optimize time use with appointment cancellations.
- Streamline referral process from primary care providers to reduce redundant appointments.
- Review patient suitability for telespecialty care.

**Equipment**

- Standardize plan for maintaining software access, reporting and troubleshooting equipment failure, and purchasing new equipment.
- Identify backup plans for care continuation during equipment or software downtime.

**Protocol**

- Standardize personnel delegation in telespecialty care.

**Training**

- Train all staff regularly on the scope and practices of telespecialty care.
- Promote awareness of telespecialty care across departments.

**Others**

- Implement a feedback system using patient and staff surveys to identify areas for improvement.
- Develop and iterate for regular communication and feedback mechanisms within the program.

### Comparison to Prior Work

Our findings are consistent with previous research, providing further support for the numerous advantages of telespecialty care for patients. In line with existing literature [7-11], this study highlights that telespecialty care offers several facilitators, including improved efficiency, convenience, and reduced travel and wait times. Telespecialty care enhances access to health care, especially for underserved areas, enabling access to specialized services [12-16] and addressing emergent conditions that patients may not have initially recognized [17]. Specifically, using store-and-forward teledermatology offers comparable effectiveness to in-person assessment, significantly reduces travel time, and expedites management [18].

Organizational barriers stemming from staffing shortages and lack of designated personnel hindered telespecialty care implementation. This barrier was exacerbated by the clinic's unmodified workflow, forcing nurses with in-person duties to take on extra work for telespecialty appointments. Consistent with our findings, a study examining the perspectives of primary care physicians on telespecialty care referral reported that teledermatology can disrupt the existing in-person workflow [13]. In situations where staffing shortages occurred, informal temporary workaround strategies were frequently used to handle exceptions to normal workflow [19]. However, reliance on workaround strategies added to the already heavy workload of staff members, as they attempted to manage the demands of telespecialty care within their existing schedules. While workarounds are commonly used in medical settings, it is important to recognize that they have the potential to increase the occurrence of medical errors [20] and place additional strain on clinics with limited resources [21].

This study highlights the criticality of establishing standardized protocols and providing ongoing training for the successful telespecialty care implementation. Stakeholders emphasized the need for protocols to guide troubleshooting and equipment failure and ensure consistent practices. These findings align with existing research, which consistently identifies limited technological knowledge, skills, and a lack of education and training as significant barriers to the implementation and acceptance of telemedicine interventions [22,23]. Furthermore, effective planning for equipment maintenance is paramount to ensure the efficient and effective provision of telespecialty care [24]. Previous research investigating the challenges of maintaining eye care equipment revealed that equipment breakdowns led to frustrating delays in conducting proper examinations and increased the risk of disease progression, resulting in poorer treatment outcomes [25]. Therefore, implementing regular maintenance protocols and establishing contingency plans are critical for minimizing disruptions and optimizing the delivery of telespecialty care.

This study reveals an increase in administrative workload for primary care providers and their support staff due to the surge in specialty care referrals. This underscores the complexities and unintended consequences of telespecialty care implementation, particularly the challenge of managing this heightened workload within limited time constraints [26]. The amplified workload pressures from specialty care referrals have compelled health care professionals to dedicate more time to collecting comprehensive patient histories for teleconsultation referrals. This additional time investment is crucial for maintaining the quality of telehealth consultations and preventing potential errors [27]. Our findings align with broader literature concerns about the workload burden imposed by administrative tasks in telehealth, emphasizing potential consequences, such as system failures, resulting from increased workload [27].

### Strengths and Limitations

This study has several limitations. First, the use of convenience sampling and unequal sample sizes across stakeholder groups may have introduced selection bias into this study. Additionally, participant perspectives were obtained solely from Teledermatology and Technology-Based Eye Care Services providers at the 2 referring sites within the VA Southeastern Network, which may not be representative of other health care settings, potentially limiting the generalizability of the findings. Future patient interviews may provide additional perspectives on telespecialty care to supplement our providers’ perspectives.

### Future Directions

Implementation of telespecialty care should apply implementation science framework to align technology, people, organizations, and context and to add value to patient care and health care systems [28]. Adapting a learning system approach that continually improves telespecialty care implementation is needed to account for health care system complexity and different user needs and to avoid unintended consequences and challenging workflow issues [28-32]. This study provided insights into the intricacies of telespecialty care implementation, shedding light on both facilitators and barriers encountered in the delivery of these services. Addressing these challenges and opportunities has the potential to increase access to care, enhance the quality of care provided, and promote the sustainability of telespecialty care innovations.

## Abbreviations

COREQ: Consolidated Criteria for Reporting Qualitative Research
VA: Veterans Affairs
